# Combined Effects of Multiple Per- and Polyfluoroalkyl Substances Exposure on Allostatic Load Using Bayesian Kernel Machine Regression

**DOI:** 10.3390/ijerph20105808

**Published:** 2023-05-12

**Authors:** Tahir Bashir, Emmanuel Obeng-Gyasi

**Affiliations:** 1 Department of Built Environment, North Carolina A&T State University, Greensboro, NC 27411, USA; 2 Environmental Health and Disease Laboratory, North Carolina A&T State University, Greensboro, NC 27411, USA

**Keywords:** PFAS, allostatic load, BKMR, combined exposures, mixture analysis

## Abstract

This study aims to investigate the combined effects of per- and polyfluoroalkyl substances (PFAS) on allostatic load, an index of chronic stress that is linked to several chronic diseases, including cardiovascular disease and cancer. Using data from the National Health and Nutrition Examination Survey (NHANES) 2007–2014, this study examines the relationship between six PFAS variables (PFDE, PFNA, PFOS, PFUA, PFOA, and PFHS) and allostatic load using Bayesian Kernel Machine Regression (BKMR) analysis. The study also investigates the impact of individual and combined PFAS exposure on allostatic load using various exposure-response relationships, such as univariate, bivariate, or multivariate models. The analysis reveals that the combined exposure to PFDE, PFNA, and PFUA had the most significant positive trend with allostatic load when it was modeled as a binary variable, while PFDE, PFOS, and PFNA had the most significant positive trend with allostatic load when modeled as a continuous variable. These findings provide valuable insight into the consequences of cumulative exposure to multiple PFAS on allostatic load, which can help public health practitioners identify the dangers associated with potential combined exposure to select PFAS of interest. In summary, this study highlights the critical role of PFAS exposure in chronic stress-related diseases and emphasizes the need for effective strategies to minimize exposure to these chemicals to reduce the risk of chronic diseases. It underscores the importance of considering the combined effects of PFAS when assessing their impact on human health and offers valuable information for policymakers and regulators to develop strategies to protect public health.

## 1. Introduction

Exposure to multiple contaminants concurrently is a fact of life [1]. Exposure to individual toxic substances, such as Perfluorooctanesulfonic acid (PFOS), has been shown to have adverse health outcomes. Yet, little is understood about how exposure to multiple contaminants affects health [2]. This is especially the case for the exposure to multiple contaminants on the stress response system, which potentially potentiates the development of chronic diseases. It is thus critical to explore the effect of multiple contaminants such as Per- and polyfluoroalkyl substances (PFAS) on stress.

PFAS have been utilized extensively in consumer goods for more than 70 years. The utilization of PFAS in commercial and industrial settings is due to their special thermal stability and surface activity characteristics, including hydro- and lipophobicity [3].

PFAS have been used as lubricants in industrial processes, as additives in pesticides and pharmaceuticals, and as lubricants in food packaging, firefighting foams, and coatings to produce nonstick and stain-resistant qualities [4]. The negative consequences of exposure to multiple PFAS could be deleterious and may have long-term effects on the life course of affected peoples [5,6].

Multiple per- and polyfluoroalkyl substances (PFAS) coexist at moderate to high levels in various situations that are also associated with high levels of chronic stress. Allostasis is a biological process that allows individuals to adapt their physiological traits to meet environmental demands and maintain physiological balance. Unlike homeostasis, which refers to the maintenance of physiological parameters within a set range, allostasis enables effective responses to adversities. However, if these challenges persist without resolution, the body may reset its physiological set points to higher levels in order to cope, potentially leading to long-term health consequences [7]. When an individual’s physiological set points are consistently reset to a higher level, they are said to be in a state of allostatic load (AL). This condition arises from ongoing physiological stress, which can result from a variety of factors, including exposure to PFAS and other stressors. The concept of allostatic load is based on the idea that repeatedly activating the hypothalamic-pituitary-adrenal (HPA) axis can have broad-ranging effects on various organ systems. These effects may include alterations in immune function, metabolic regulation, and cardiovascular health, among others. Therefore, understanding and mitigating the impact of stressors on allostatic load is crucial for maintaining overall health and well-being [7,8,9,10].

Allostatic load combines multiple physiological markers to create a comprehensive index of biological stress, which can quantify the impact of chronic exposure to stressors on the body. Psychosocial stressors, such as poverty, inequality, and lack of resources, can increase allostatic load in communities, interacting with environmental conditions. Health outcomes are influenced by various contextual factors, including regional policies and infrastructure. Real-world exposure to stressors, such as PFAS and other environmental contaminants, varies considerably at individual and population levels [11,12]. Given the complex and varied nature of real-world exposures to stressors, the combined risk of different exposures is not yet fully understood. However, data analytics approaches offer a promising avenue for studying the impact of these exposures on stress indicators such as allostatic load. By combining multiple sources of data, such as environmental monitoring and health records, these approaches can help identify and evaluate the impact of multiple stressors on human health. Ultimately, a better understanding of the combined risks of different exposures will be essential for developing effective strategies to mitigate their impact and improve overall health outcomes [13].

Bobb et al. introduced a Bayesian Kernel Machine Regression (BKMR) for evaluating pollutant mixtures using the R software, version 4.1.2 (R Foundation for Statistical Computing, Vienna, Austria) [14]. BKMR, implemented in the “bkmr” package for R, is a versatile tool used in toxicology, epidemiology, and other fields to estimate the health impacts of pollutant mixtures. It uses hierarchical variable selection and Gaussian predictive methods to model the health outcomes of mixtures under the kernel function, while controlling for potential confounding factors [14,15]. By accurately estimating the health impacts of pollutant mixtures, BKMR has the potential to inform effective interventions to mitigate their negative effects on human health [16]. In the context of evaluating pollutant mixtures, procedures like BKMR can effectively address the issue of collinearity between mixture components and assess their overall health effects [17].

BKMR modeling is a powerful method that offers several advantages over traditional modeling approaches. Firstly, it enables thorough modeling of both chemical exposure and outcome, allowing for a more accurate understanding of the relationships between these variables. Additionally, BKMR modeling assesses chemical components independently of the independent—dependent function, providing a clearer picture of the individual effects of each chemical. Another key advantage is its ability to assess chemical mixture effects, which is particularly important in situations where exposure to multiple chemicals is common [15,17].

BKMR is a useful tool for assessing the health effects of chemical mixtures, such as multiple PFAS. It employs variable selection to estimate the importance of each exposure in the mixture through posterior inclusion probabilities (PIPs). This approach helps overcome challenges associated with collinearity and a high correlation between exposures in epidemiological and toxicological studies, making BKMR a valuable tool for researchers [13,18].

Through the use of BKMR, this study hypothesizes that there exists a significant association between exposure to multiple PFAS simultaneously and high levels of allostatic load. The primary objectives of this study are to determine the extent of association between exposure to multiple PFAS and allostatic load, identify the specific PFAS that contribute the most to the observed association, and investigate these factors by modeling allostatic load as a categorical and continuous variable.

The findings of this study provide critical insights into the effects of multiple PFAS exposure on human health and contribute to the public health literature aimed at understanding and reducing the risk of PFAS exposure. Furthermore, identifying specific PFAS that contribute the most to the observed association can help prioritize regulatory actions and interventions aimed at reducing exposure to these harmful chemicals.

## 2. Materials and Methods

### 2.1. Description of Cohort

This investigation utilized data from the National Health and Nutrition Examination Survey (NHANES), administered by the Centers for Disease Control and Prevention (CDC). The NHANES collected biological specimens and survey data on demographics, social, and clinical factors for the civilian, non-institutionalized population residing in any of the 50 states of the United States of America and the District of Columbia (DC) using a complex, multistage probability design. This study used data for adults aged 20 years and older.

For this study, data were assessed over four cycles: 2007–2008, 2009–2010, 2011–2012, and 2013–2014. The final sample size for our study was *n* = 23,482 individuals, consisting of 11,414 males and 12,068 females. The study aimed to investigate several variables of interest, including exposure to specific perfluoroalkyl substances (PFAS), such as Perfluorodecanoic acid (PFDE), Perfluorononanoic acid (PFNA), Perfluorooctanesulfonic acid (PFOS), Perfluoroundecanoic acid (PFUA), Perfluorooctanoic acid (PFOA), and Perfluorohexanesulfonic acid (PFHS). Additionally, the study also examined the allostatic load index, which was operationalized using the following clinical and biomarkers: Systolic Blood Pressure (SBP), Diastolic Blood Pressure (DBP), triglycerides, HDL cholesterol, total cholesterol, C-reactive protein (CRP), Body Mass Index (BMI), hemoglobin A1C, albumin, and creatinine clearance. Overall, this study utilized a large and diverse sample size, as well as a comprehensive set of variables of interest, to investigate the relationships between PFAS exposure and allostatic load.

### 2.2. Blood Serum Measurements

As part of the NHANES, participants visited a mobile examination center (MEC) where they provided blood samples for laboratory analysis. The blood samples were used to assess a wide range of health indicators, including markers of exposure to environmental contaminants such as per- and polyfluoroalkyl substances (PFAS).

To ensure that laboratory measurements were conducted under comparable circumstances, the MEC’s controlled environment was used. Blood samples were collected in polypropylene and polyethylene containers, and depending on the participant’s age, at least 0.5 mL of serum was processed. Samples were then frozen or refrigerated before being sent to labs across the country.

For this study, participants who were scheduled for a morning session were required to fast for nine hours before the blood draw. After the initial blood draw, they were instructed to ingest 75 g of dextrose (10 oz of glucose solution) within ten minutes. Two hours later, a second blood sample was collected.

### 2.3. PFAS Extraction and Quantitation

To extract and quantify PFAS from human serum, a 50 L aliquot was first diluted with formic acid and injected into a commercial column switching system for concentration on a solid-phase extraction column. This method of extraction was used to separate the analytes from other serum constituents. High-performance liquid chromatography was then employed to separate the analytes from each other. Detection and quantification of the PFAS was achieved using tandem mass spectrometry, which converts liquid phase ions into gas-phase ions. To detect and quantify the PFAS, an electrospray ionization source was modified to produce negative-ion Turbo Ion Spray (TIS) ions. This method has a low limit of detection (LOD) in the low parts per billion range, which enables the rapid detection of these PFAS in human serum. Overall, this method provides a reliable and sensitive approach for the detection and quantification of PFAS in human serum.

All PFAS analytes in the dataset had a constant detection limit of 0.10 ng/mL. For each analyte, two variable names were provided in NHANES: a value of “0” indicated that the result was at or above the detection limit, while a value of “1” indicated that the result was below the detection limit. In cases where the analytic result was below the lower limit of detection, an imputed fill value was inserted into the analyte results field. This value was calculated as the lower limit of detection divided by the square root of 2 (LLOD/sqrt(2)), which was 0.10/√2 = 0.07. Consequently, the LOD for each PFAS was either 0.10 or 0.07 [19,20].

### 2.4. Quantifying Allostatic Load

The markers of interest that went into the allostatic load were: Systolic blood pressure—SBP, diastolic blood pressure—DBP, triglycerides, high-density lipoprotein (HDL) cholesterol, and total cholesterol, inflammatory (C-reactive protein—CRP), and the metabolic systems (body mass index—BMI, hemoglobin A1C, albumin, and creatinine clearance and they were quantified as follows. The NHANES procedure for obtaining blood pressure involved collecting a maximum of 3 brachial systolic and diastolic BP readings for each participant using appropriate cuff sizes based on midarm circumference. Trained medical personnel followed a standard protocol and used a Baumanometer true gravity mercury wall model at the MEC. Triglycerides and HDL-C were measured using the Roche modular P chemistry analyzer (Roche Diagnostics, Indianapolis, IN, USA). CRP was measured via latex enhanced nephelometry and quantified using anti-CRP antibodies with a hydrophilic shell and polystyrene core of CRP particles. BMI was calculated as weight (kg) divided by the square of standing height (m^2^), while glycohemoglobin was measured using the A1c G7 HPLC Glycohemoglobin Analyzer (Tosoh Medics, Inc., San Francisco, CA, USA). The Roche/Hitachi Modular P Chemistry Analyzer was used to measure urine creatinine (Roche Diagnostics, Indianapolis, IN, USA). Finally, a non-competitive, double-antibody method was employed for measuring human urinary albumin, involving covalently attaching an antibody to human albumin to polyacrylamide beads, reacting the solid-phase antibody with a urine specimen, and measuring the fluorescence of the resulting complex using a fluorometer.

### 2.5. Operationalizing Allostatic Load

Influenced by earlier research [2,21,22,23,24,25], the study operationalized allostatic load as a cumulative index of physiological dysfunction in the cardiovascular, inflammatory, and metabolic systems. This index included multiple markers, such as triglycerides, high-density lipoprotein (HDL) cholesterol, total cholesterol, and systolic and diastolic blood pressure, along with other measures like body mass index, hemoglobin A1C, albumin, and creatinine clearance. Quartiles were used to divide the markers based on their distribution within the dataset. Specifically, the top quartile was considered high-risk for all markers except for albumin, creatinine clearance, and HDL cholesterol, for which the lowest 25% of the distribution was deemed to be the highest risk based on prior literature [26,27,28,29,30,31,32]. Each study participant was given a value of 1 if they were in the high-risk category or a value of 0 if they were in the low-risk category for each marker which was then added up to get a total allostatic load value out of 10. Using a high-score criterion, we divided the allostatic load scores into two categories [33]. An allostatic load greater than or equal to 3 was required for a score to be considered high; otherwise, it was considered low.

### 2.6. Statistical Analysis

This study aimed to investigate the impact of six per- and polyfluoroalkyl substances (PFAS)—PFDE, PFNA, PFOS, PFUA, PFOA, and PFHS—on allostatic load, which was the dependent variable. Allostatic load, a measure of physiological dysregulation, was assessed using established biomarkers. The six PFAS were considered the independent variables, and we aimed to model their combined effect on allostatic load through statistical analysis. This study employed BKMR analysis to explore the relationship between the independent and dependent variables and assess the magnitude and significance of their associations.

#### 2.6.1. Statistical Results Analysis Using BKMR

##### BKMR Analysis

We used BKMR with the hierarchical variable selection method due to highly correlated variables and collinearity of key variables within the dataset. In this study we built BKMR models in the R program via the R package (bkmr). BKMR models evaluated the role of mixtures or multipollutant exposures (e.g., PFOA, PFOS, etc.) on allostatic load using the kmbayes function.

#### 2.6.2. BKMR Modeling for the Binary Outcome (Allostatic Load as 0 and 1)

Using BKMR we modeled the effect of multiple PFAS on allostatic load (as a categorical variable) adjusting for covariates “Gender”, “Age”, “Cigarette smoking”, “Physical activity”, “Ethnicity”, “Occupation”, “Income”, “Alcohol Consumption”, “Education”, “Birthplace”, and “Time in the US” in our primary analysis and then in subsequent analysis adding lead (Pb) to the covariates in order to account for the demonstrated association of blood lead levels with allostatic load.

Binary outcomes were included in the BKMR package using the probit model via a general linear model for efficiency and convenience of computation and to overcome some of the issues that may arise in the dataset, such as collinearity under Bayesian inference [14,34]. Finally, we modeled allostatic load as a continuous variable to see if the associations held.

## 3. Results

### 3.1. Critical PFAS Values Elevating Allostatic Load Is US Adults

The overall analytical plan for the first part of this study was to assess the combined effect of PFDE, PFNA, PFOS, PFUA, PFHS, and PFNA on allostatic load (as a categorical variable) while adjusting for the following covariates: Gender, Age, Cigarette smoking, Physical activity, Ethnicity, Occupation, Income, Alcohol Consumption, Education, Birthplace, and Time in the US.

**Figure 1:**
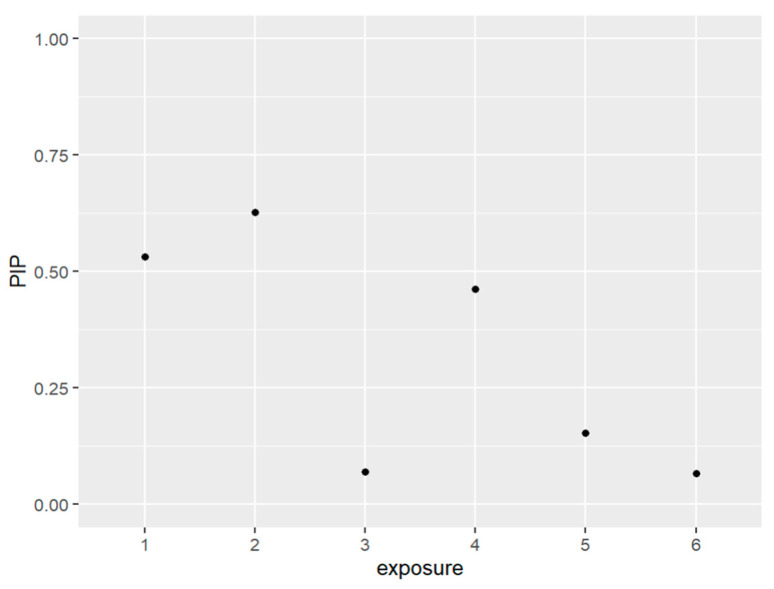
The PIPs—Posterior inclusion probabilities—for each exposure in the model. PIPs values closest to 1 are the most important, and values closest to 0 are the least important and not included in the model. Note: exposure numbers are as follows: 1 for PFDE, 2 for PFNA, 3 for PFOS, 4 for PFUA, 5 for PFOA, and 6 for PFHS.

Figure 1 and Table 1 below present the PIPs which measure the percentage of the data that backs the inclusion variables in the model. In other words, it quantifies a variable’s importance to be included within the model. The exposures that were included in our model based on their posterior inclusion probabilities (PIPs) values were PFDE, PFNA, and PFUA.

The posterior inclusion probabilities (PIPs) of selected variables in the model range from 0 to 1, where a higher value indicates a stronger association with the outcome variable, which is allostatic load in this study. PIPs close to 1 suggest that the variables are important predictors of allostatic load and play a significant role in the model. Conversely, variables with PIPs closer to 0 are less relevant to the outcome of interest and do not impact allostatic load significantly. These results are presented in Table 1.

The study firstly compares different models (BKMR, Oracle, Linear, and True) and finds that the BKMR model is suitable for analyzing multipollutant exposures. The BKMR model provides a better fit than other models for both individual variables and the overall model, as shown in Table 1.

**Figure 2:**
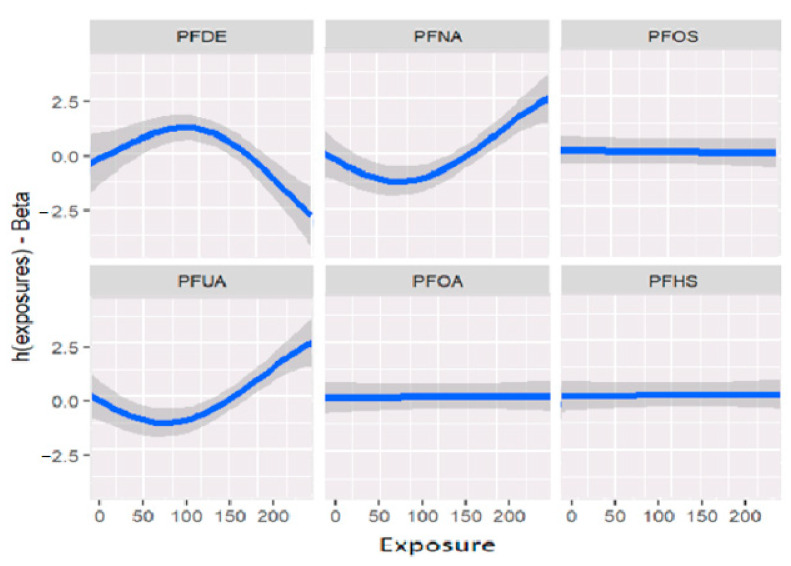
Estimation of exposure-response as univariate relation on the effects of the outcomes and health status when all remaining exposures are at their 50th percentiles and the covariates are fixed constant.

Figure 2 displays the univariate relationship between the allostatic load variable and each individual PFAS included in the model, while holding constant the other exposures at their median values and the covariates at fixed levels. The analysis reveals that some of the PFAS variables did not show significant associations with the outcome allostatic load. Notably, the results suggest that exposures to PFDE, PFNA, and PFUA are significantly associated with allostatic load, while other variables did not show significant associations.

Figure 2 and Table 2 demonstrate the relationships between exposures and the outcome variable (allostatic load) after adjusting for covariates (previously described as confounders). These associations reveal that certain exposures, such as PFDE, PFNA, and PFUA, are linked to allostatic load, with varying levels of impact indicated by the steepness of the incline in the figure. The uphill and downhill sections of the figure represent high and low levels of exposure, respectively. For example, PFDE has a mean value of 2.40 for high allostatic load and −4.00 for low allostatic load, while PFHS has a mean value of 1.10 for high allostatic load and −1.03 for low allostatic load. These values increase or decrease based on the exposure levels and the impact of cofactors that affect the exposure.

**Figure 3:**
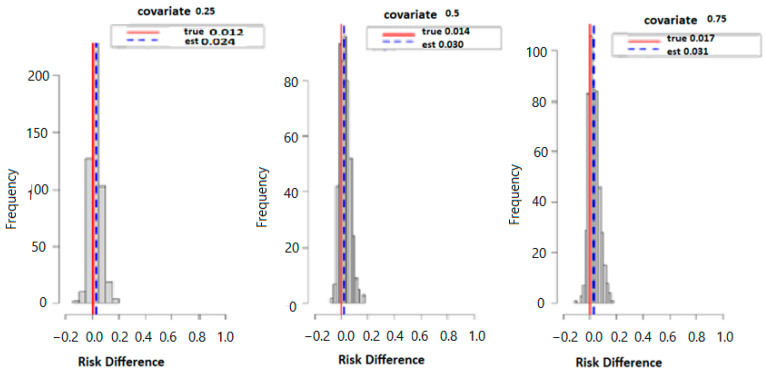
The true model displays the overall risk difference (RD) of posterior distribution and the evaluation of posterior mean estimate (est.) and true RD by fixing the exposure at the 25th, 50th, and 75th percentiles with fixed covariate values at the same percentiles.

Figure 3 below displays the results of probit regression modeling with risk difference (RD) inferred from the output. Here we calculated the conditional RD comparing the probability of allostatic load when the second exposure was set to its 75th percentile versus its 50th percentile (with other exposures at their median value), for exposures fixed at their 25th, 50th, and 75th percentiles. Overall, the estimation (est) point and true values were 0.012 and 0.024 for the 25th, 0.014 and 0.030 for the 50th, and finally, 0.017 and 0.031 for the 75th percentile. Overall, the data suggests that higher exposure to multiple PFAS increases the risk of higher allostatic load.

### 3.2. BKMR Modeling for the Binary Outcome (Allostatic Load as 0 and 1) with Lead (within the Covariates)

The modeling below was repeated with lead (Pb) added as a covariate to see if the association between allostatic load and blood lead levels significantly altered the outcomes of the study.

**Figure 4:**
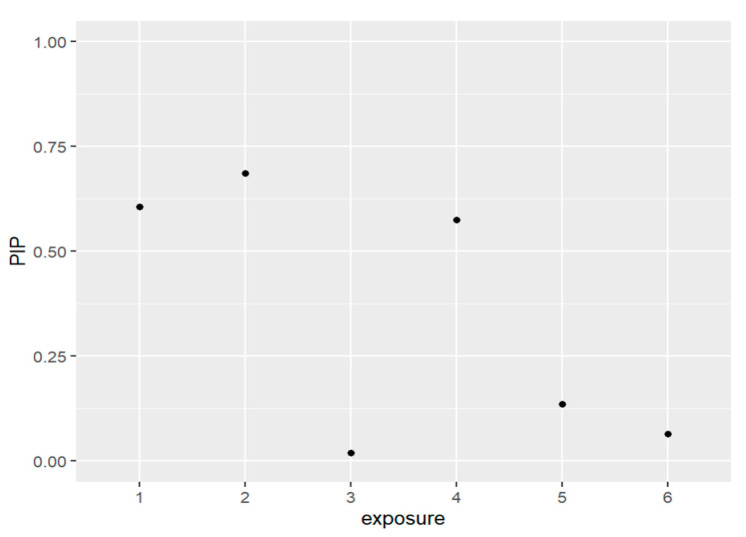
The PIPs—Posterior inclusion probabilities—for each exposure in the model and PIPs values close to 1 that indicate the most important, and values close to 0 are the least important and not included in the model. Note: exposure numbers are as follows: 1 for PFDE, 2 for PFNA, 3 for PFOS, 4 for PFUA, 5 for PFOA, and 6 for PFHS.

Figure 4 and Table 3 below present the PIPs for the exposures. The exposures to be included in the model based on the resulting PIP values are PFDE, PFNA, and PFUA. These PFAS are the same variables that were included in the prior models which did not have lead (Pb) as a covariate.

**Figure 5:**
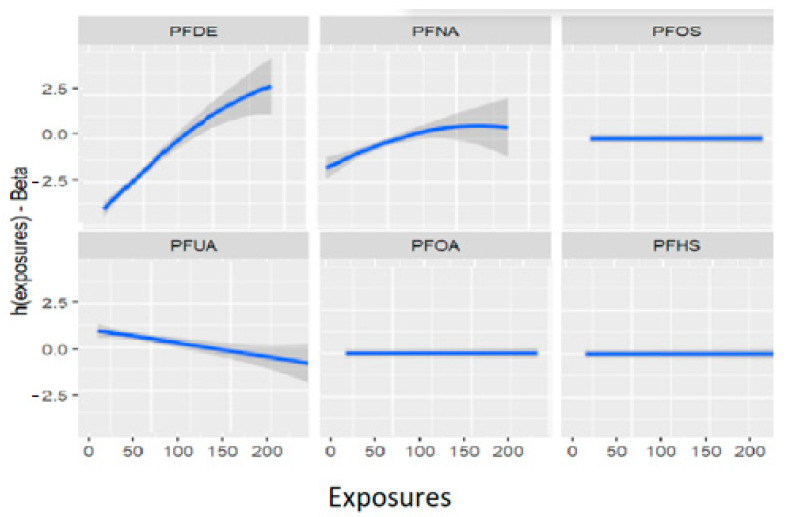
Estimation of exposure-response as univariate relation on the effects of individual PFAS on allostatic load when all other PFAS are at their 50th percentiles and the study covariates are fixed (constant).

Figure 5 displays the univariate association between allostatic load and the PFAS of interest using the BKMR model, while the other exposures are held constant at their median values and the covariates are fixed. The figure demonstrates that PFOS, PFOA, and PFHS have no significant association with allostatic load (when modeled as a binary variable), while PFDE, PFNA, and PFUA have significant associations. These associations are presented after adjusting for covariates, including lead (Pb). Table 4 provides further details on these associations, showing the mean values of each PFAS for high and low allostatic load. The relationships between the exposures and allostatic load vary in terms of their steepness, indicating different levels of exposure. The values can either increase or decrease depending on the amount of exposure and the impact of cofactors. The uphill and downhill sections of Figure 5 and Table 4 represent high and low levels of exposure, respectively, and illustrate these relationships. Overall, these results highlight the complex associations between PFAS exposures and allostatic load, which can be further explored using multivariate models.

**Figure 6:**
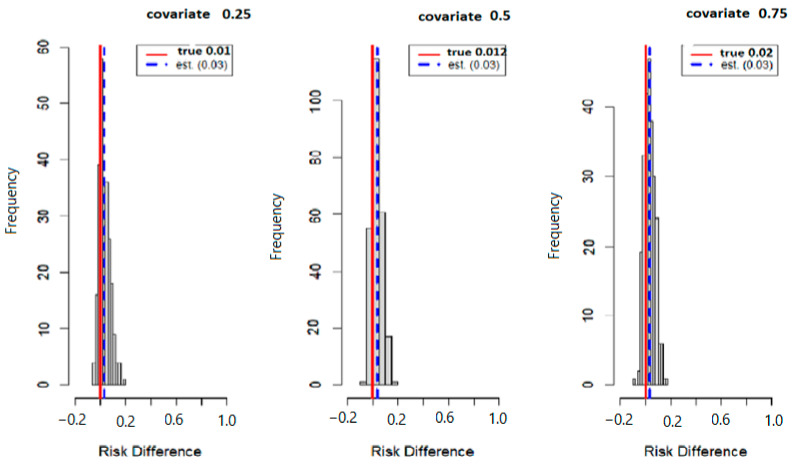
True model displays overall risk difference (RD) of posterior distribution and the evaluation of posterior mean estimate (est.) and true RD by fixing the exposure at the 25th, 50th, and 75th percentiles with fixed covariate values at the same percentiles.

Figure 6 below displays the results of probit regression modeling with risk difference (RD) inferred from the output. Here we calculated the conditional RD comparing the probability of allostatic load when the second exposure was set to its 75th percentile versus its 50th percentile (with other exposures at their median value), for exposures fixed at their 25th, 50th, and 75th percentiles. Overall, the estimation (est.) point and true values were 0.01 and 0.03 for the 25th percentile, 0.012 and 0.030 for the 50th percentile, and 0.02 and 0.03 for the 75th percentile.

### 3.3. BKMR Modeling for the Continuous Outcome (Allostatic Load as Countinuous Variable)

To investigate the relationship between multiple PFAS and allostatic load, we modeled allostatic load as a continuous variable rather than a binary variable, as the latter approach could have missed important nuances in the relationship. We employed the Bayesian Kernel Machine Regression method to evaluate the impact of multiple PFAS exposures on allostatic load, while controlling for covariates such as gender. This approach allowed us to comprehensively analyze the associations between PFAS and allostatic load and examine potential nonlinear and interactive effects. The models were adjusted for relevant covariates to ensure the accuracy and reliability of the results.

**Figure 7:**
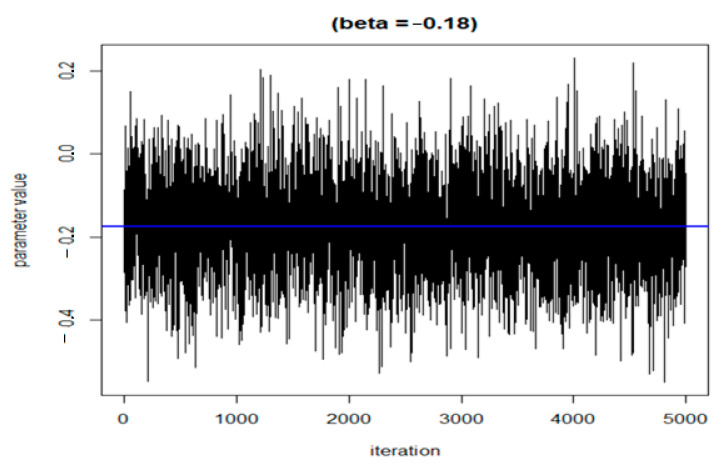
Change in the beta value (h) in the model when the sample was run.

**Figure 8:**
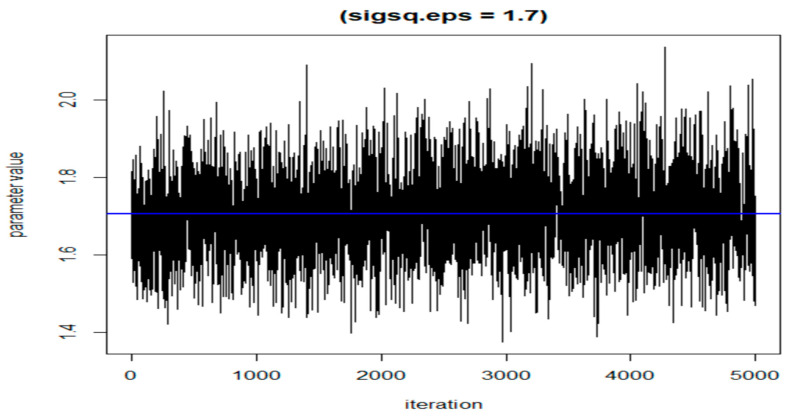
The value of epsilon (eps) during the dataset simulation run.

**Figure 9:**
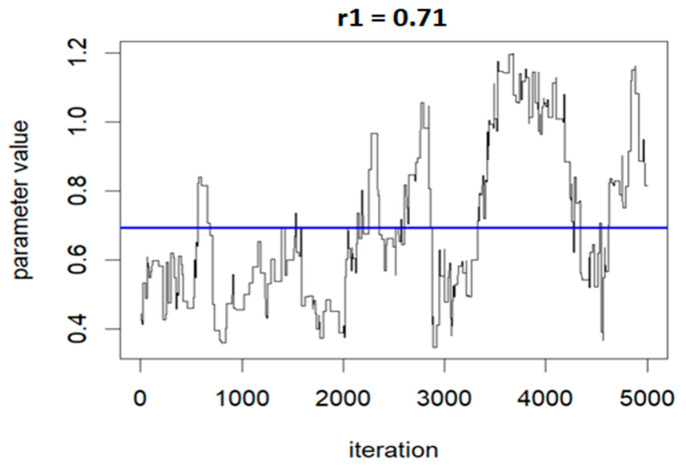
Correlation (r) value showing the relationship changes during the model run: r1 = 0.71 for PFNA and allostatic load.

The following three figures (Figure 7, Figure 8 and Figure 9) illustrate the variations of parameters/values throughout the sample runs. Figure 7 displays the beta1 (first exposure) value, which is equal to −0.18. Beta1 was selected to represent the sample relationship, and the figure demonstrates how the relationships between study variables change throughout the simulation runs, from zero to the end (5000 iterations in this case). Figure 8 depicts an average epsilon (ε)—(sigsq.eps) value of 1.7, which measures the error of the true regression line. Lastly, Figure 9 displays the correlation value (r1), which is 0.71. r1 reflects the relationship between PFNA and allostatic load and how the parameters change when the model is executed. Table 5 shows all the r1 values from the models.

**Figure 10:**
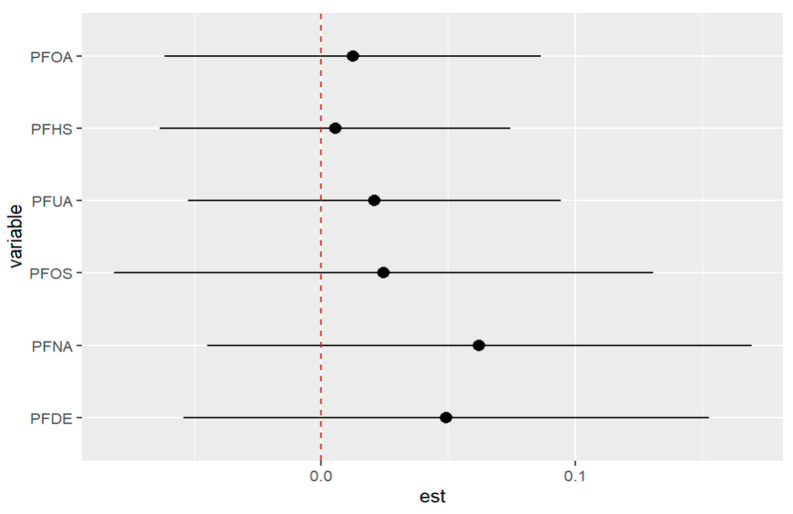
The outcome of a single pollutant when interacting with other pollutants comparing when the 25th percentile is compared to the 75th percentile when fixed. Note: “est” is the estimation of the allostatic load value when the PFAS percentile is changed from the 25th to the 75th percentile.

Table 6 displays the results of a univariate analysis of the effect of PFAS exposures on allostatic load modeled as a continuous variable. The table presents the PIP values for each PFAS, with PFDE, PFNA, and PFOS having the highest values and thus selected for the model. The remaining exposures had little to no association with the outcome (allostatic load), as indicated by their small PIP values. The analysis involved assessing the impact of a variable at different percentiles while fixing all other variables and the covariate at a certain percentile. For instance, when exposure to PFDE changes from the 50th to the 75th percentile, allostatic load decreases by −0.409 units with all other exposures fixed at their 75th percentile. The numbers in Table 6 and Figure 10 below show how the study variables interact, highlighting the effects of single-variable outcomes when other variables are fixed at different percentiles. The values demonstrate the association between PFAS exposure and allostatic load, indicating varying effects at different percentiles. The last column shows the overall risk values of changes in a single pollutant when all other pollutants are fixed and compared from their 25th to 75th percentiles.

Table 7 presents the concentration distribution of PFAS across different cycles. It shows the lower quartile, median, upper quartile, and interquartile range of PFDE, PFNA, PFOS, PFUA, PFOA, and PFHS for each year from 2007 to 2014. The table demonstrates that the data is similar and equivalent across the various cycles, which provides context to the study results.

For example, in 2007–2008, the lower quartile, median, and upper quartile concentrations of PFDE were 0.070, 0.090, and 0.200, respectively. The interquartile range was 0.130, indicating that the middle 50% of the data was between 0.025 and 0.155. The same pattern can be observed for the other PFAS variables in each cycle.

Overall, Table 7 provides valuable information on the distribution of the PFAS data, which is important for understanding the study results and drawing conclusions.

**Figure 11:**
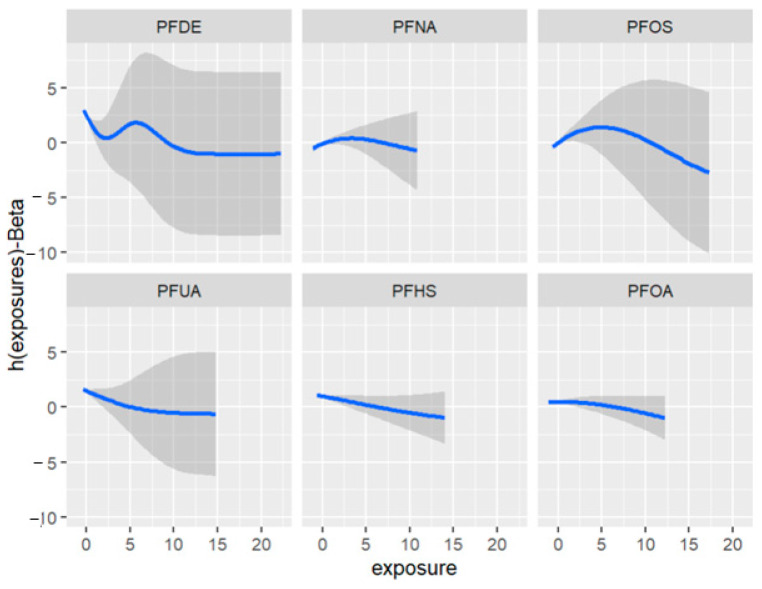
Univariate association between individual exposure and the outcome –while other exposures are fixed to their 50th percentile and cofounder is fixed (constant). Note: the exposures to PFAS (i.e., PFDE, PFNA, PFOS, PFUA, PFHS, and PFOA) concentrations are reported in (ng/mL).

Figure 11 depicts the univariate exposure-response relationship, where each individual PFAS and its association with allostatic load are shown while fixing the remaining exposures to their median (50th percentile), as presented in Table 6 above. The covariate, gender, is held constant in this analysis. The figure illustrates the associations between exposures and the allostatic load after adjusting the model for gender as a covariate. For example, the PFDE exposure is associated with a steeper increase in allostatic load, indicating varying levels of exposure, and the uphill trend in the figure represents a higher level of exposure. This suggests that the values of allostatic load increase with increasing levels of PFDE exposure.

**Figure 12:**
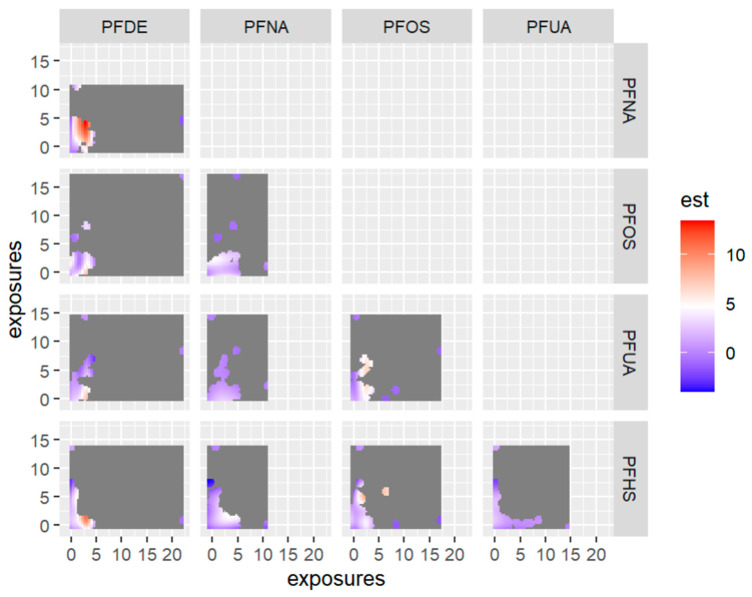
Contour images or plots for bivariate exposure-response relationships only estimated for points that are within 0.5 units from an observed data point because we specified that only 0.5 should be displayed. Points further than this distance are grayed out in the plot. Note: the exposures to PFAS (i.e., PFDE, PFNA, PFOS, PFUA, PFHS) concentrations are reported in (ng/mL). Note: “est” is the estimation value after bivariate or 2-way interactions between PFAS of interest.

Figure 12 displays the bivariate relationship between 2-way PFAS exposures and allostatic load, focusing on pairs of PFAS—allostatic load interactions while fixing the remaining exposures at their 50th percentile (median). This approach was taken based on the association of PFAS with allostatic load and the use of PIPs. In this analysis we excluded any exposure not associated with the outcome in this bivariate relationship.

**Figure 13:**
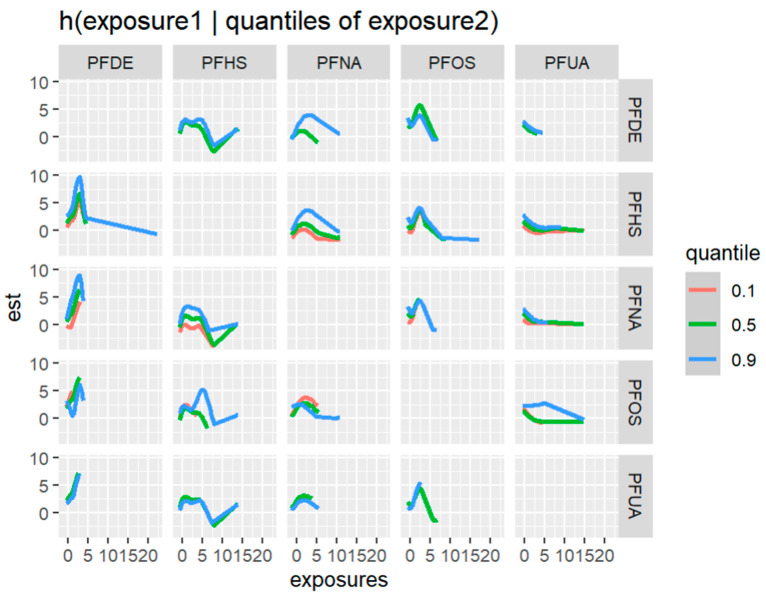
Bivariate exposure-response association showing one exposure (predictor or independent), another exposure fixed at different quantities (i.e., 10th, 50th, 90th percentiles), and all other independent exposures are fixed at different quantities (i.e., 10th, 50th, 90th percentiles). Note: “est” is the estimation value after bivariate or 2-way interactions between PFAS of interest. The exposures to PFAS (i.e., PFDE, PFHS, PFNA, PFOS, and PFUA) concentrations are reported in (ng/mL).

Figure 13 depicts an investigation of bivariate or 2-way interactions, which examines the relationship between two exposures and their corresponding response variables. This analysis examines the effect of one exposure on the response variable while holding the other exposure fixed at different percentiles (namely, the 10th, 50th, and 90th percentiles).

**Figure 14:**
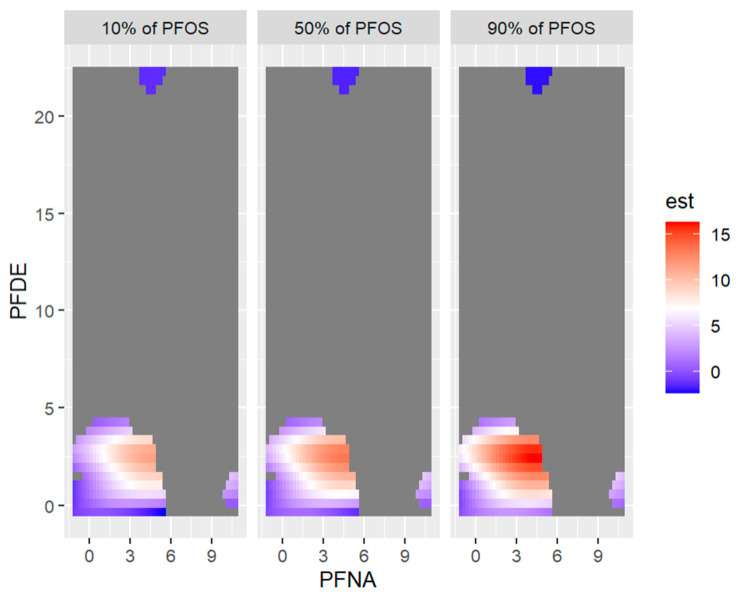
Bivariate exposure-outcome function of PFNA and PFOS for PFDE fixed at either its 10th, 50th, or 90th percentile, and for all other exposures are fixed at their 50th percentile values. Note: the exposures to PFAS (i.e., PFDE, PFNA, PFOS) concentrations are reported in (ng/mL).

Figure 14 below shows the visualization of 2-exposures-response or three-way interactions by fixing the third variable (exposure) at the 10th, 50th, and 90th percentiles.

**Figure 15:**
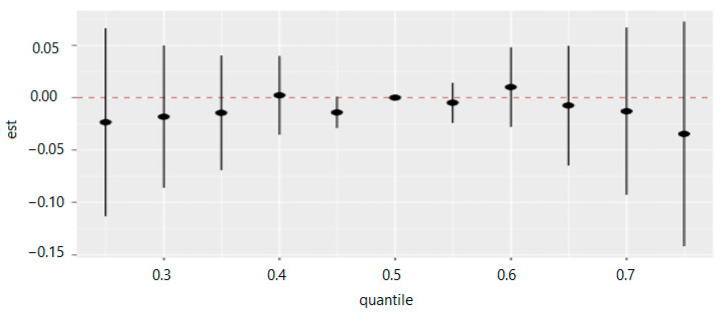
Summary of overall health effects of the exposures (multimixers) on the outcome depends on various percentiles (form 25th to 75th percentiles). Note: “est” is the estimation value of multivariable effects on allostatic load (e.g., the estimation of overall effects of all exposures on allostatic load is 0.009 when are fixed on the 60th percentile as compared to when all of them are at the 50th percentile (median)).

Table 8 and Figure 15 measure the total effect of all exposures or mixtures. The exposures are fixed at different quantities starting from the 25th percentile to 75th percentile at increments of 5 using the 50th percentile (median) to compare the exposures. The estimation for all exposures at the 50th percentile shown at zero (red dashed line) demonstrates that when all exposures are fixed at the 75th percentile, the allostatic load decreases by 0.035 units, and for the 40th percentile, the allostatic load increases by 0.002 units.

Figure 15 demonstrates the overall summary of the effect of multiple PFAS on allostatic load. The collective degrees across the PFAS and allostatic load both increase and decrease simultaneously. In general, the overall summary of the exposure effects demonstrates that allostatic load decreases both with increases and decreases of the percentiles in and around the 50th percentile, with some differences in a direction depending on the degrees of rising or diminishing.

**Figure 16:**
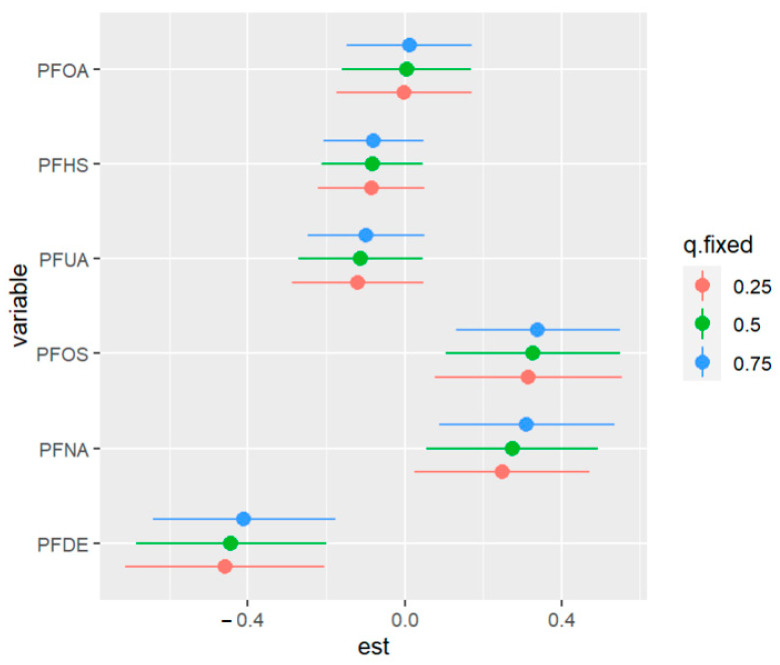
Univariate exposure of health effects at 95% confidence intervals comparing the changes in each exposure from the 25th, 50th, and 75th percentiles when the remaining exposures are fixed at the similar percentiles. Note: “est” is the estimation value of the univariate effects of PFAS on allostatic load.

Figure 16 displays the impact of an individual PFAS exposure on allostatic load, while holding the remaining exposures fixed at other percentiles. This approach enables us to assess the contribution of each PFAS exposure to the overall risk. The figure shows that higher levels of PFOS and PFNA are correlated with a higher allostatic load. In addition, the associations of PFDE and PFNA with allostatic load increase when some exposures increase from the 25th to the 75th percentile. Conversely, when other exposures (such as PFHS) decrease in value from the 25th to the 75th percentile, the association of PFOS with allostatic load increases. These findings suggest the possibility of interactions among some of the exposures, such as the interactions of PFOA with PFDE and PFUA with PFOS. Overall, this analysis sheds light on the complex relationships among PFAS exposures and their impact on allostatic load

## 4. Discussion

In this study, we used BKMR to assess the relationships between allostatic load and six PFAS combinations in a sample of US adults from the NHANES. The concentration of various PFAS were relatively homogenous across the four cycles of data making the data ideal for our study. Specifically, the findings from the study reveal important insights into the concentrations and time trends of the PFAS of interest in the study cohort. The results indicate that the concentrations of PFAS were relatively stable over time, with only small variations observed.

BKMR modeling is a statistical technique that is employed to evaluate the significance of variables based on their posterior inclusion probability, and the multivariable relationships between the predictor variables and outcome variables, including univariate, bivariate, and multivariate exposure-response relationships, as well as their effects on one another. This method was used to identify the most important PFAS and their interactions in producing high allostatic load, while accounting for potential confounders and covariates. By considering the relationships between multiple variables simultaneously, BKMR modeling enabled a more comprehensive understanding of the complex interplay between the exposures and outcomes. Among the PFAS examined in this study, BKMR analysis revealed that higher serum concentrations of PFDE, PFNA, and PFUA tended to be associated with a higher allostatic load with these PFAS being identified as the most important in the relationship between PFAS and allostatic load. When allostatic load was modeled as a continuous variable the most critical PFAS were PFDE, PFOS, and PFNA. The fact that PFOS became more important in the analysis suggests that the nature of the data and the underlying relationship between allostatic load and PFAS is complex. To best capture nonlinear interactions between the variables, modeling allostatic load as continuous may paint a more accurate picture of the effects of multiple PFAS. This is so because allostatic load modeled as a continuous variable has more granularity to detect subtle changes, and as a categorical variable allostatic load only provides information on the category to which each observation belongs, and not on specific values within the category. Finally, as a continuous variable, allostatic load has increased sensitivity to changes or variations in data.

This study is the first of its kind to model PFAS with allostatic load using BKMR. That said, other studies may offer additional insight. Ding et al. conducted a study to examine the association between PFAS exposure and adipocytokine concentrations and found significant correlations between PFAS mixtures and the health-related outcomes of leptin and adiponectin in their models. Specifically, the PFAS chemicals PFDA, PFOS, PFNA, and PFHpS were identified as important contributors to these associations [35]. Tian et al. studied the link between PFAS exposure and folate levels using BKMR. They found that PFOA interacted with PFOS, PFNA, and PFDA to negatively affect RBC folate, with the negative impact increasing as PFOA levels rose. The mixture of five PFAS was inversely associated with RBC folate overall [36]. Other studies using BKMR analysis have shown adverse effects of multiple PFAS and liver injury [37], cardiovascular disease [38] and kidney dysfunction (glomerular filtration rate) [39] among others. These findings together speak to the plausibility of our hypothesis in the context of the results we obtained.

In our study, using BKMR’s feature of being able to estimate hierarchical importance, PFNA was found to have a larger influence on allostatic load, followed by PFDE and PFUA when modeling allostatic load as a binary variable. Such insight can be critical for public health officials working in environments where the PFAS exposure is vast and contains various PFAS. By considering all exposures in a multivariable space and connecting the exposure with the health outcome via the Gaussian kernel function, the BKMR model allows for an excellent exploration of the relationship between PFAS and allostatic load.

The BKMR model’s conclusions about the direct relationship between PFNA, PFDE, and PFOS with allostatic load when modeled as a continuous variable are interesting in that the most critical variables in order from high to low were PFOS, PFDE, and PFNA. Thus, when allostatic load is viewed as a continuous variable the traditional PFAS, PFOS, becomes the most important variable. This is especially insightful since BKMR modeling correct for the confounding effect, which is brought on by the strong correlations between PFAS when compared to traditional multiple linear regression models [2]. Specifically, BKMR deals with either high correlations among multiple PFAS or non-linear relationships and thus captures associations which may have been missed in traditional models.

PFAS compounds in combination with one another may interact and produce a cumulative effect that is either more or less than the total of the fractions of the individual PFAS because people are typically exposed to numerous PFAS at once from various sources [40]. The mechanisms by which the interactions occur between PFAS to produce allostatic load need to be further investigated to confirm our findings. Despite not performing mechanistic studies, we hypothesized that combined exposure to PFAS may increase allostatic load through promoting oxidative stress and diminishing antioxidant systems i.e., via GSH depletion, increasing blood pressure and cholesterol, and promoting an obesogenic state. This hypothesis is based on research that has occurred using animal models and in vitro [40,41,42].

The strengths of this study are as follows. The study used mixture analysis to evaluate the combined exposure of multiple PFAS on allostatic load. In addition, we used a nationally representative sample of adults. Finally, the study adjusted for the relevant confounders which lowered the bias that may have occurred. The cross-sectional study design, one of the study’s drawbacks, makes it challenging to conclude whether a causal relationship between PFAS exposure and allostatic load exists. Finally, we did not employ the NHANES survey weights in BKMR models, comparable to other earlier studies using NHANES data, because weighting algorithms were not included in the R packages for BKMR.

## 5. Conclusions

In the current investigation, we found direct correlations between exposure to selected PFAS and allostatic load. The PFAS mixtures showed statistically significant overall direct correlations with allostatic load. Our study demonstrated that exposure to particular PFAS in combination might adversely promote the body’s stress response.

Based on the results of the current study, there are significant associations between allostatic load and exposure to PFDE, PFNA, PFOS, and PFUA. Our findings suggest that an accumulation of PFAS exposure could have negative effects on the body’s ability to cope with stress.

## Figures and Tables

**Table 1:** PIP and comparison of four models (BKMR, Oracle, Linear, and True) examining PFAS effects on allostatic load.

			Overall effects			
			Models			
			BKMR ^1^	Oracle ^2^	Linear ^3^	TRUE ^4^
			0.172	−0.239	−0.315	−0.429
			Individual effect			
			Models			
Variable number	Variable	PIP ^5^	BKMR	Oracle	Linear	TRUE
1	PFDE	0.532	0.085	−1.341	−0.729	−1.629
2	PFNA	0.627	0.018	0.421	0.139	0.272
3	PFOS	0.071	0.001	−0.728	0.035	0.062
4	PFUA	0.463	0.031	0.154	−0.281	−0.441
5	PFOA	0.153	0.002	1.153	−0.011	−0.014
6	PFHS	0.067	0.001	−0.357	−0.036	−0.064

Note: ^1^ is Bayesian Kernel Machine Regression (BKMR). ^2^ is the Oracle model that is used as a glm (generalized linear model). ^3^ is the linear model. ^4^ is the True model using all variables with no adjustment. ^5^ is PIP (posterior inclusion probability), which quantifies the importance of the variable in the variable selection.

**Table 2:** The high and low values of allostatic load for PFAS variables after the adjustment.

Allostatic Load	PFAS					
	PFDE *N* = 21,597	PFNA *N* = 22,405	PFOS *N* = 23,200	PFUA *N* = 20,856	PFOA *N* = 23,482	PFHS *N* = 19,952
	Mean	Mean	Mean	Mean	Mean	Mean
High	2.40	3.00	1.02	4.20	1.04	1.10
Low	−4.00	2.30	−0.90	−2.00	−0.81	−1.03

**Table 3:** PIP and comparison of four models (BKMR, Oracle, Linear, and True) assessing the effects of PFAS on allostatic load.

			Overall effects			
			Models			
			BKMR ^1^	Oracle ^2^	Linear ^3^	TRUE ^4^
			0.162	−0.078	−0.183	−0.429
			Individual effect			
			Models			
Variable Number	Variable	PIP ^5^	BKMR	oracle	Linear	TRUE
1	PFDE	0.606	0.067	−1.384	−0.768	−1.629
2	PFNA	0.687	0.023	0.429	0.144	0.272
3	PFOS	0.021	0.001	−0.699	0.034	0.062
4	PFUA	0.574	0.048	0.085	−0.291	−0.441
5	PFOA	0.135	0.002	1.201	−0.009	−0.014
6	PFHS	0.064	0.001	−0.411	−0.034	−0.064

Note: ^1^ is Bayesian Kernel Machine Regression (BKMR). ^2^ is the Oracle model that is used as a glm (generalized linear model). ^3^ is the linear model. ^4^ is the True model using all variables with no adjustment. ^5^ is PIP (posterior inclusion probability), which quantifies the importance of the variable in variable selection.

**Table 4:** The high and low values of allostatic load for PFAS variables after the adjustment.

Allostatic Load	PFAS					
	PFDE *N* = 21,597	PFNA *N* = 22,405	PFOS *N* = 23,200	PFUA *N* = 20,856	PFOA *N* = 23,482	PFHS *N* = 19,952
	Mean	Mean	Mean	Mean	Mean	Mean
High	4.80	2.45	0.06	1.85	0.07	0.01
Low	−3.91	−2.5	−0.03	−2.00	−0.02	−0.03

**Table 5:** Correlation values between PFAS and AL.

	AL	PFDE	PFNA	PFOS	PFUA	PFOA	PFHS
AL	1	0.80	0.71	0.74	0.06	0.52	0.27
PFDE	0.80	1	−0.23	0.05	−0.041	0.09	0.21
PFNA	0.71	−0.03	1	−0.08	0.01	−0.09	0.19
PFOS	0.74	0.56	0.91	1	0.75	0.53	0.47
PFUA	0.06	0.16	0.45	0.53	1	0.84	0.56
PFOA	0.52	0.76	0.84	0.92	0.57	1	0.48
PFHS	0.27	0.18	0.07	0.30	0.63	0.49	1

**Table 6:** PIP values for each exposure and the changes in allostatic load when the exposures are fixed in selected percentiles.

Variable Number	Variable	PIP ^a1^	Individual Exposure Fixed nth Percentile ^a2^			Percentiles Changed ^a3^
			25th	50th	75th	25th to 75th
			Effects Changed on the Outcome			
1	PFDE	0.959	−0.458	−0.442	−0.409	0.0493
2	PFNA	0.581	0.248	0.274	0.311	0.062
3	PFOS	0.985	0.315	0.326	0.330	0.025
4	PFUA	0.446	−0.120	−0.112	−0.099	0.021
5	PFHS	0.294	−0.115	−0.111	−0.076	0.006
6	PFOA	0.180	0.008	0.031	0.035	0.012

Note: ^a1^ PIP is posterior inclusive probability for the exposures. ^a2^ is univariable or single-independent variable health risk on the response when each of the exposures is fixed at the 25th, 50th and 75th percentile or the change effect value. ^a3^ single-variable health risks when on the response variable by comparing the changes on each exposure for its 25th to its 75th percentiles or the result of subtracting the value of 25th percentiles from 75th percentiles.

**Table 7:** PFAS with their lower quartile, the median, upper quartile, and interquartile range concentrations.

Years	Quartiles	Variables					
		PFDE	PFNA	PFOS	PFUA	PFOA	PFHS
2007–2008	Q1	0.070	0.058	0.140	0.070	0.070	0.070
	Q2	0.090	0.058	0.150	0.110	0.320	0.940
	Q3	0.200	0.902	12.300	0.140	3.500	1.800
	IQR	0.130	0.844	12.160	0.070	3.430	1.730
2009–2010	Q1	0.070	0.058	0.140	0.070	0.070	0.070
	Q2	0.083	0.058	0.140	0.096	0.107	0.370
	Q3	0.200	0.984	6.500	0.100	2.700	1.050
	IQR	0.130	0.926	6.360	0.030	2.630	0.980
2011–2012	Q1	0.070	0.058	0.140	0.070	0.070	0.070
	Q2	0.102	0.058	0.140	0.092	0.542	0.414
	Q3	0.160	0.670	4.850	0.100	1.600	0.980
	IQR	0.090	0.612	4.710	0.030	1.530	0.910
2013–2014	Q1	0.070	0.058	0.140	0.070	0.070	0.070
	Q2	0.101	0.058	0.140	0.110	0.253	0.531
	Q3	0.200	0.550	0.250	0.125	0.790	0.950
	IQR	0.130	0.492	0.110	0.055	0.720	0.880

Note: Q1 or lower quartile corresponds with the 25th percentile. Q2 (the median), corresponds with the 50th percentile. Q3 or the upper quartile corresponds with the 75th percentile. The interquartile range (IQR), it is defined as the difference between the 75th and 25th percentiles of the data (IQR = Q3 − Q1).

**Table 8:** Multivariable effects on allostatic load when the exposures are fixed at different quantities started from the 25th percentile to the 75th percentile at increments of 5 using the median values to compare the effects.

All Exposures Fixed at nth Percentile *	Est Effects on Outcome **
25th	−0.024
30th	−0.018
35th	−0.015
40th	0.002
45th	−0.014
50th	0.000
55th	−0.005
60th	0.009
65th	−0.008
70th	−0.013
75th	−0.035

Note: * is showing the percentiles (nth) from the 25th to the 75th that all the exposures are fixed to. ** are the estimated point values for all of the exposures together on the health effects.

## Data Availability

The NHANES dataset is publicly available online, accessible at cdc.gov/nchs/nhanes/index.htm (accessed on 5 February 2023).
